# Initial Safety and Feasibility of Steerable Ureteroscopic Renal Evacuation: A Novel Approach for the Treatment of Urolithiasis

**DOI:** 10.1089/end.2021.0759

**Published:** 2022-08-24

**Authors:** Roger L. Sur, Shashank Agrawal, Brian H. Eisner, George E. Haleblian, Arvind P. Ganpule, Ravindra B. Sabnis, Mahesh R. Desai, Glenn M. Preminger

**Affiliations:** ^1^ Department of Urology, UC San Diego Health, San Diego, California, USA.; ^2^ Department of Urology, Muljibhai Patel Urological Hospital, Nadiad, India.; ^3^ Division of Urology, Department of Surgery, Massachusetts General Hospital, Newton Wellesley Hospital, Boston, Massachusetts, USA.; ^4^ Department of Surgery, Harvard Medical School, Brigham and Women's Hospital, Boston, Massachusetts, USA.; ^5^ Division of Laparoscopy and Robotic Surgery, Department of Urology, Muljibhai Patel Urological Hospital, Nadiad, India.; ^6^ Division of Urologic Surgery, Duke University, Durham, North Carolina, USA.

**Keywords:** urolithiasis, aspiration, evacuation, ureteroscopy, SURE

## Abstract

**Background:**

There is a need to reliably render urolithiasis patients completely stone free with minimal morbidity. We report on the initial safety and feasibility with steerable ureteroscopic renal evacuation (SURE) in a prospective study using basket extraction as a comparison.

**Materials and Methods:**

A pilot randomized controlled study was conducted comparing SURE with basket extraction postlaser lithotripsy. SURE is performed using the CVAC™ Aspiration System, a steerable catheter (with introducer). The safety and feasibility of steering CVAC throughout the collecting system under fluoroscopy and aspirating stone fragments as it was designed to do were evaluated. Fluoroscopy time, change in hemoglobin, adverse events through 30 days, total and proportion of stone volume removed at 1 day, intraoperative stone removal rate, and stone-free rate (SFR) at 30 days through CT were compared.

**Results:**

Seventeen patients were treated (*n* = 9 SURE, *n* = 8 Basket). Baseline demographics and stone parameters were not significantly different between groups. One adverse event occurred in each group (self-limiting ileus for SURE and urinary tract infection for Basket). No mucosal injury and no contrast extravasation were observed in either group. The CVAC catheter was steered throughout the collecting system and aspirated fragments. There was no significant difference in fluoroscopy time, procedure time, change in hemoglobin, or stone removal rate between groups. SURE removed more and a greater proportion of stone volume at day 1 *vs* baskets (202 mm^3^ *vs* 91 mm^3^, *p* < 0.01 and 84% *vs* 56%, *p* = 0.022). SURE achieved 100% SFR at 30 days *vs* 75% for baskets, although this difference was not statistically significant (*p* = 0.20).

**Conclusions:**

This initial study suggests SURE is safe, feasible, and may be more effective in stone removal postlaser lithotripsy compared to basketing. More development is needed, and larger clinical studies are underway.

## Introduction

The primary goal for urolithiasis treatment is to maximize stone removal and minimize patient morbidity.^1^ Residual fragments (RF) were once thought to be clinically insignificant, but evidence shows that RF are associated with a 20% to 43% rate of stone events, including pain, stone regrowth, infection, emergency department visits, hospital admissions, and additional procedures.^2–6^ Current procedures, including extracorporeal shockwave lithotripsy (SWL), percutaneous nephrolithotomy (PCNL), and ureteroscopy (URS), have achieved significant advances, and yet efficient, reliable, and complete stone removal remain an elusive goal.

We hypothesized that stone fragments after URS laser lithotripsy could actively be eliminated if aspiration could be safely and easily applied throughout the collecting system. In this study, we report on the initial safety and feasibility of steerable ureteroscopic renal evacuation (SURE), a new, minimally invasive treatment for urolithiasis.

## Patients and Methods

### Study protocol

A pilot randomized controlled study was conducted between December 2018 and February 2019 at Muljibhai Patel Urological Hospital (MPUH) in Nadiad, India. The protocol (EC/502/2018) was approved by the Institutional Review Board, and participants provided written informed consent before enrollment. It was conducted in accordance with the World Medical Association Declaration of Helsinki and all amendments and the International Conference on Harmonization Guideline for Good Clinical Practice.

Eligible patients were ≥18 years of age with a single renal stone 5 to 15 mm in diameter or multiple stones (all ≤15 mm in diameter) on kidney, ureter, and bladder radiograph and/or noncontrast helical CT. Patients were excluded if they had ureteral calculi, urologic anatomic/congenital abnormalities, confirmed pregnancy, active or untreated infection, or prior surgical ipsilateral treatment within the prior 3 months.

Four experienced endourologists participated in the trial after SURE training in a porcine and/or a bench top, silicon kidney model with collapsible calyces to simulate CVAC device navigation and the SURE mechanism of irrigation and aspiration. This study was designed to evaluate SURE compared to standard basket stone extraction, but not powered for statistical comparison as this was a pilot study used as the basis for planning larger future studies. All patients received standard URS laser lithotripsy and were then randomized to undergo stone extraction (1) through SURE using the CVAC™ Aspiration System (SURE group) (Calyxo, Inc., Pleasanton, CA) or (2) using a zero-tip basket (Basket group). Both patients and radiologists reading CT scans were blinded to the stone removal method.

### Study objectives

The primary safety objective was to evaluate the safety (Clavien-Dindo classification system and modified Satava classification) of the SURE procedure and use of the CVAC Aspiration System through 30 days of follow-up. Fluoroscopy time and change in hemoglobin were also measured and compared. The primary feasibility objective was to demonstrate the ability to steer the CVAC catheter throughout the collecting system in the SURE group through fluoroscopy and aspirate stone fragments up to 2 mm in size.

Secondary objectives were to compare effectiveness between the SURE and Basket groups, including the following: total and proportion of stone volume removed intraoperatively (both measured at postoperative day 1 through CT), intraoperative stone removal rate, and stone-free rate (SFR) through 30-day CT.

Since 3D reconstruction CT scanning was not available, stone volume was calculated using formulas for a prolate ellipsoid (*π*/6 × *a* × *b* × *b*) for stones <9 mm maximum diameter and an oblate ellipsoid (*π*/6 × *a* × *a* × *c*) for stones 9 to 15 mm maximum diameter, where *a* is the equatorial diameter, *b* is the polar diameter, and *c* is the third measurable diameter.^7^

### SURE procedure and CVAC aspiration system

**FIG. 1.**
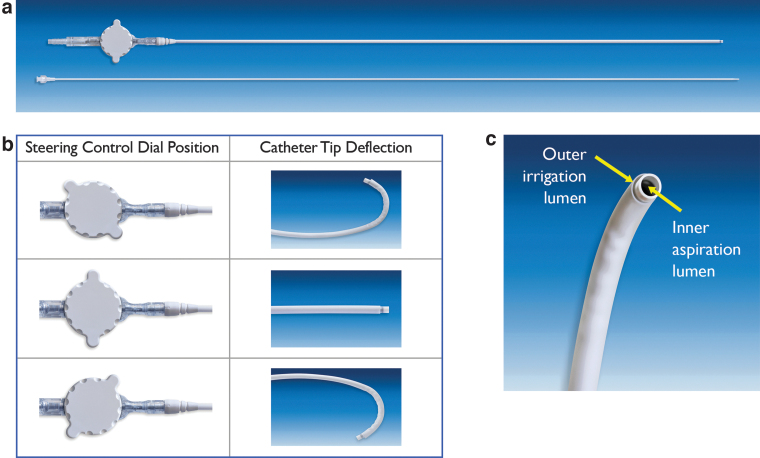
**(a)** CVAC™ Aspiration Catheter and Introducer; **(b)** CVAC Aspiration Catheter steering control dial enables tip deflection; **(c)** CVAC Catheter distal tip showing dual lumen design.

The SURE procedure is performed using the CVAC Aspiration System (Calyxo, Inc.), a custom aspiration catheter (with introducer) designed to navigate to all areas of the renal collecting system under fluoroscopic guidance (Fig. 1a). In this first-generation device, the CVAC catheter has a working length of 70 cm and an outer diameter of 11.9F. The control handle has a steering control dial that allows the operating surgeon to steer and deflect the tip of the device into all parts of the collecting system (Fig. 1b). The control handle also enables, on demand, irrigation through an irrigation port connected to a fluid line and syringe. Intermittent irrigation is applied using a 10 cc syringe. Aspiration is achieved by attaching the vacuum port on the CVAC catheter to standard operating room wall/machine suction set to 150 to 200 mm Hg.

On the control handle, suction may be switched on and off using the vacuum controller. The catheter shaft has a dual-lumen design that allows for simultaneous irrigation and aspiration of stone fragments up to 2.5 mm and for a rapid transition of fluid flow directions within the collecting system (Fig. 1c). The inner aspiration channel has an internal diameter of 7.5F (2.5 mm).

### Surgical technique

In all patients, rigid cystoscopy with retrograde pyelogram was performed through a 5F open-ended catheter, followed by passage of a 0.035-inch gauge wire. A 12F/14F ureteral access sheath was then passed over the working wire. A single-use digital flexible ureteroscope was used to perform URS with holmium laser lithotripsy. Lithotripsy was performed using a 270-micron laser fiber with laser settings determined by surgeon preference (range 0.6–1.0 J, 6–12 Hz) and the assigned stone removal arm. For the SURE group, the operating surgeon attempted to fragment all stones to ≤2 mm.

The Basket group underwent stone fragment extraction using a 1.9F zero-tip nitinol basket until no further fragments could be basketed and the operating surgeon determined the patient was stone free based on endoscopic inspection.

**FIG. 2.**
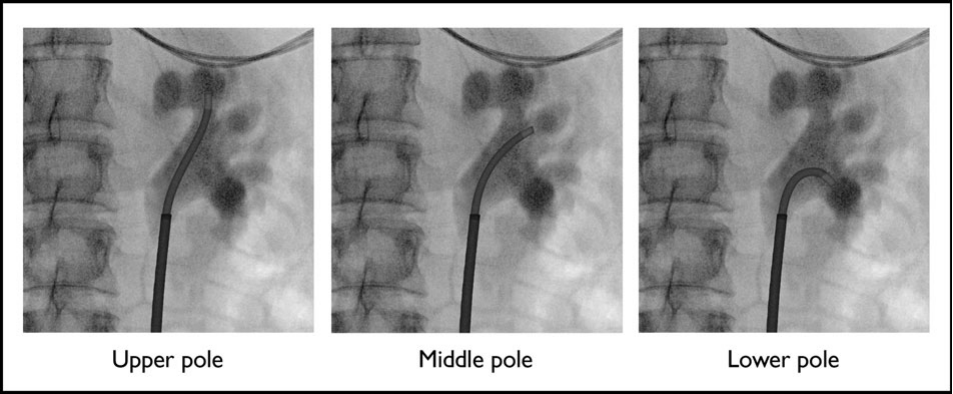
CVAC Aspiration Catheter in upper, middle, and lower pole under fluoroscopy (as depicted in porcine model).

In the SURE group, the following steps were performed: (1) the ureteroscope and laser fiber were removed from the access sheath; (2) the CVAC catheter and introducer were inserted over the guidewire through the access sheath; (3) after removing the introducer, the tip of the CVAC catheter was navigated under fluoroscopy into each calyx starting in the upper pole and working toward the lower pole (Fig. 2); and (4) in each calyx, stone fragments and dust were evacuated by alternating irrigation and suction. Typically, two full “sweeps” (defined as applying irrigation and aspiration throughout the collecting system, moving from the upper pole calyces to the middle calyces and then to the lower calyces and renal pelvis) were performed.

The CVAC catheter was then removed, and the ureteroscope was reintroduced. Ureteroscopic evaluation was performed to assess any RF. If necessary, the CVAC aspiration system was reintroduced, and SURE continued until the surgeon determined that the patient was endoscopically and fluoroscopically stone free. No extraction method cross-over was allowed, and retrieval baskets were not used in any patient in the SURE group.

In both cohorts, final endoscopic inspection along with a repeat retrograde pyelogram was performed to document mucosal injury and/or extravasation of contrast. A 6F JJ ureteral stent was placed at the end of the procedure. Noncontrast CT was performed postoperatively at days 1 and 30 and independently read by MPUH radiologists blinded to randomization. Being stone free was defined as zero RF.

### Statistical analysis

All endpoints were analyzed based on patients treated per protocol. Safety and feasibility outcomes were tabulated and reported in full. Comparative endpoints were analyzed using a Student's *t*-test for continuous variables and chi square test for categorical variables. Probability values <0.05 were regarded as statistically significant.

## Results

Seventeen patients were treated and included in the final analysis (*n* = 9 SURE, *n* = 8 Basket). Initially, 19 patients were randomized (*n* = 11 SURE, *n* = 8 Basket). For this per-protocol analysis, data from two patients were censored: one because of a protocol deviation (intraoperative diverticulum and large stone burden exceeding the inclusion/exclusion criteria) and one attributable to significant intrarenal clots associated with a pre-existing perforation discovered at the time of URS. No adverse event was observed in either of the censored patients. There were no significant differences in demographics or preoperative stone parameters between the two groups (Table 1).

**Table 1.** Baseline Patient Characteristics (Mean)

	SURE group (*n* = 9)	Basket group (*n* = 8)	*p*
Age, years	42	37	0.25
% Males	88	62	0.2
BMI	22.3	23.4	0.8
% Single stone	55	50	0.81
% Lower pole stone	78%	63%	0.49
Baseline stone volume, mm^3^	267	210	0.55
Stone density, HU	786	926	0.4

BMI = body mass index; SURE = steerable ureteroscopic renal evacuation.

The primary safety objective was achieved: no serious adverse event was observed in either group. There was one patient with self-limiting ileus (Clavien-Dindo Grade I) in the SURE group and one urinary tract infection (Clavien-Dindo Grade II) in the Basket group (Table 2). No mucosal injury was observed intraoperatively, and no contrast extravasation was seen in any patient in either group through ureteroscopic evaluation and retrograde pyelogram at the end of the procedure. There was no endoscopic intraoperative complication experienced to report using the modified Satava classification. There was no significant difference in hemoglobin change between groups (0.8 g/dL SURE *vs* 0.6 g/dL Basket, *p* = 0.28). There was also no significant difference in fluoroscopy time or procedure time (measured from the start of cystoscopy to the end of stent placement) between groups (Table 3).

**Table 2.** Complications According to Clavien-Dindo Classification

Grade	Complication	SURE group,* n/N *(%)	Basket group,* n/N *(%)
I	Ileus	1/9 (11.1%)	0/8 (0%)
II	UTI	0/9 (0%)	1/8 (12.5%)

UTI = urinary tract infection.

**Table 3.** Perioperative Outcomes

	SURE group (*n* = 9)	Basket group (*n* = 8)	*p*
Volume of stone removed at postoperative day 1, (mm^3^)	202 ± 94 (64–318)	91 ± 42 (33–140)	<0.01
% Stone volume removed at postoperative day 1, (% of baseline)	84 ± 19 (37–100)	56 ± 24 (21–88)	0.022
Stone-free rate based on 30-day CT scan	100%	75%	0.20
Fluoroscopy time, seconds	318 ± 120 (184–600)	295 ± 213 (125–673)	0.78
Procedure time, minutes	54 ± 17 (30–80)	39 ± 22 (15–75)	0.13
Stone removal rate (mm^3^/min)	16.6 ± 9.5 (6.0–35.9)	9.6 ± 6.4 (2.0–22.0)	0.099

Mean ± standard deviation (range).

**FIG. 3.**
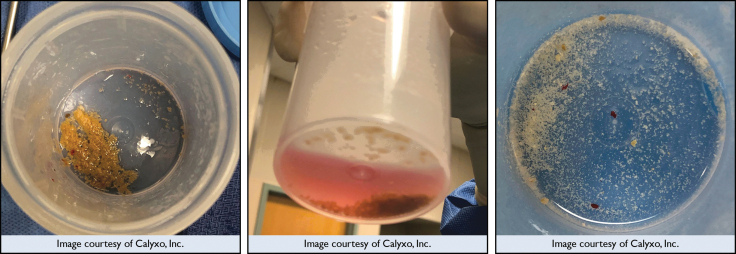
Representative stone debris collected from three patients after SURE procedure. SURE, steerable ureteroscopic renal evacuation.

The feasibility endpoint was also achieved: the CVAC catheter was successfully navigated throughout the renal collecting system in each patient and successfully aspirated stone fragments and dust (Fig. 3).

SURE removed a significantly greater stone volume and significantly greater proportion of total stone volume at day 1 when compared with basket extraction (202 mm^3^ *vs* 91 mm^3^, *p* < 0.01 and 84% *vs* 56%, *p* = 0.022, Table 2). The higher proportion of stone volume removed led to a 100% SFR as measured by CT at 30 days for SURE *vs* 75% for Basket (*p* = 0.20), although this difference was not statistically significant.

In a subset analysis of patients in this study with lower pole stones (SURE, *n* = 7 and Basket, *n* = 5), significantly more stone volume was removed with SURE (188 mm^3^ *vs* 72 mm^3^, *p* = 0.038) and the rate of stone removal was also faster (14.9 mm^3^/min *vs* 7.1 mm^3^/min, *p* = 0.039, Table 3). The difference in proportion of stone removed was not different for SURE *vs* Basket (80% *vs* 65%, *p* = 0.28). For those five patients without lower pole stones, there were three with renal pelvis stones only (SURE, *n* = 1 and Basket, *n* = 2) and two with ureteral stones only (SURE, *n* = 1 and Basket, *n* = 1).

For these patients, generally, the volume and percentage of stone removed at postoperative day 1 was greater in the SURE group than in the Basket group, although the sample sizes were too low to determine statistical significance (Table 4).

**Table 4.** Perioperative Patient Outcomes by Stone Location

	Lower pole		Renal pelvis only		Ureter only	
	SURE (*n* = 7)	Basket (*n* = 5)	SURE (*n* = 1)	Basket (*n* = 2)	SURE (*n* = 1)	Basket (*n* = 1)
Volume of stone removed at postoperative day 1, (mm^3^)	188 ± 103 (64–298)	72 ± 37 (32–128)	221	116 ± 32 (93–139)	278	139
*p*-Value	*p* = 0.038		NA^a^		NA^a^	
% Stone volume removed at postoperative day 1, (% of baseline)	80 ± 21 (37–100)	65 ± 25 (28–88)	100	52 ± 4.7 (49–55)	90	21
*p*-Value	*p* = 0.28		NA^a^		NA^a^	
Stone removal rate (mm^3^/min)	14.9 ± 6.8 (5.8–24.2)	7.1 ± 3.0 (2.1–9.6)	9.5	9.7 ± 8.2 (4.0–15.5)	35.9	22.0
*p*-Value	*p* = 0.039		NA^a^		NA^a^	

Mean ± standard deviation (range).

^a^ NA, not applicable (sample size too low to calculate).

## Discussion

This initial human study demonstrates that SURE is safe and feasible for the removal of stone fragments after URS laser lithotripsy. Four experienced endourologists completed their first SURE procedures with safety outcomes similar to basketing. Although the study was not powered for statistical comparison, the results suggest that SURE may be more effective than basket extraction at fragment removal, with a significantly higher percentage and absolute amount of stone volume removed (Table 3). Another promising finding was that SURE achieved 100% SFR at 30 days compared to 75% for the Basket group, although this difference was not statistically significant.

The SFR for SWL ranges from 68% to 90% for ureteral stones and requires multiple attempts (1.11–1.76 procedures) to achieve these results.^8^ URS SFR outcomes range from 62% to 85.6%.^9,10^ Even in a study of aggressive basket extraction in which every attempt to remove all stone fragments after fragmentation was undertaken (requiring an average of 44 and range of 1–164 device passes), the SFR was 55% (based on noncontrast CT ∼8 weeks postprocedure).^11^ PCNL employs more invasive and direct access to the kidney compared to URS and would seem to afford a higher corresponding SFR. However, in a large global PCNL study, the 30-day SFR was 76%, and 15% of patients required additional treatment.^12^

This review underscores the shortcomings of contemporary urolithiasis treatment in achieving straightforward, reliable, efficient, and complete stone removal. Although the small sample size in our study does not support a definitive comparison with published SFR, we note that the SFR in the Basket group is consistent with the literature and find the 100% SFR in the SURE group to be promising.

We also obtained CT imaging at postoperative day 1 to isolate the immediate effect of the SURE procedure without the added benefit of spontaneous passage that would be seen in delayed imaging (e.g., postoperative day 30). We acknowledge the additional ionizing radiation added with this methodology, and this ethical decision was balanced with its intended benefit.

Although aspiration has been used before in urolithiasis procedure, to our knowledge, this is the first time it has been applied from the transurethral approach with a steerable catheter that can enter each calyx for target aspiration of stone fragments. Aspiration has been used in PCNL and through URS rigid sheaths, but in these instances, the delivery devices are rigid, obviating the benefit of aspiration with difficulty in accessing all areas where stone treatment is needed. This study demonstrated that delivering aspiration through the unique steerable, surgeon-controlled CVAC catheter is feasible and promising toward more effective and complete stone fragment removal.

This initial study was also encouraging in demonstrating safety of the SURE procedure. We found no evidence of mucosal injury, bruising, or edema and no contrast extravasation in any patient. Also, fluoroscopy time and thus radiation exposure risk were not significantly different between groups, although we acknowledge it may be higher than an average URS procedure given the high stone burden of the enrolled patients. Steering the CVAC catheter under fluoroscopy appeared to be consistent with the standard technique of placing guidewires, access sheaths, and ureteroscopes. Even so, investigation into developing a system that is steered under direct visualization is a future consideration.

One theoretical concern was the potential for clogging of lumen of the CVAC device with stone fragments during aspiration. We did not experience any instance of clogging in this study, but one could envision this scenario. Hence, bench testing was performed and demonstrated that hydraulic cycling of the vacuum lumen using a 10 cc syringe can effectively clear a clogged device. Another concern is that this first-generation CVAC aspiration system requires a 12F/14F access sheath, which is a limitation for some patients.

As to be expected with a pilot safety and feasibility study, limitations include the small sample size, a first-generation CVAC system, a still-evolving SURE technique, and the Investigators' lack of prior human clinical experience with SURE. The study was not powered for statistical comparison as this was the initial study used to plan for larger future studies. Thus, the statistical analyses and subset analyses of patients by stone location (lower pole, renal pelvis only, and ureter only) must be viewed with discretion in this context.

Future studies are planned with larger sample sizes, refined techniques, and CVAC device improvements to further understand how effectively SURE can remove stone fragments efficiently and render patients stone free reliably. Another limitation is that ellipsoid formulas were used to calculate stone volume, which is not as accurate as 3D modeling. In future studies, we will seek to work with facilities that have 3D reconstruction CT scanning capabilities.

Even with this small feasibility study, it was encouraging to find that SURE has the potential to improve access and stone removal for all patients included in the study *and* in the subset analysis of patients with lower pole stones. In the subset of patients with lower pole stones, SURE removed more stone volume and was significantly faster in fragment elimination compared to basket extraction. As it is well known that lower pole stone location may be challenging (in terms of operative time, SFR, ureteroscope damage), SURE may have the potential to deliver substantial health economic savings in reduced operating time, reduced scope damage, and improved patient outcomes through more efficient and effective stone removal.^13–15^ Future device and procedure development will continue to work toward this goal.

## Conclusions

SURE is a new URS approach to postlaser lithotripsy stone removal that allows for aspiration to safely be applied throughout the entire collecting system. In this pilot safety and feasibility study, we have shown the procedure is safe, is feasible, and has the potential to remove stones more efficiently and effectively compared to standard of care. There are still many challenges to work through including device and technique refinements. Further study of this novel treatment is warranted, and larger studies are underway.

## Abbreviations Used

BMI: body mass index
CT: computed tomography
HU: Hounsfield units
MPUH: Muljibhai Patel Urological Hospital
PCNL: percutaneous nephrolithotomy
RF: residual fragments
SFR: stone-free rate
SURE: steerable ureteroscopic renal evacuation
SWL: extracorporeal shockwave lithotripsy
URS: ureteroscopy, ureteroscopic
UTI: urinary tract infection

## References

[1] Holst D, Bechis S, Zupkas P, et al. Minimally invasive percutaneous nephrolithotomy: Initial North American experience. J Endourol 2021;25:596–600. 10.1089/end.2020.0574 33050718

[2] Raman J, Bagrodia A, Gupta A, et al. Natural history of residual fragments following percutaneous nephrostolithotomy. J Urol 2009;181:1163–1168. 19152935 10.1016/j.juro.2008.10.162

[3] Rebuck D, Macejko A, Bhalani V, Ramos P, Nadler R. The natural history of renal stone fragments following ureteroscopy. Urology 2011;77:564–569. 21109293 10.1016/j.urology.2010.06.056

[4] Chew B, Brotherhood H, Sur R, et al. Natural history, complications and re-intervention rates of asymptomatic residual stone fragments after ureteroscopy: A report from the EDGE Research Consortium. J Urol 2016;195:982–986. 26585680 10.1016/j.juro.2015.11.009

[5] Emmott A, Brotherhood H, Paterson R, Lange D, Chew B. Complications, re-intervention rates, and natural history of residual stone fragments after percutaneous nephrolithotomy. J Endourol 2018;32:28–32. 29037066 10.1089/end.2017.0618

[6] Sorokin I, Canvasser N, Lay A, Antonelli J, Pearle M. Natural history of residual fragments confirmed by computed tomography after ureteroscopy. J Urol 2018;199(4S):PD45-02.

[7] Finch W, Johnston R, Shaida N, Winterbottom A, Wiseman O. Measuring stone volume—Three-dimensional software reconstruction or an ellipsoid algebra formula? BJU Int 2014;113:610–614. 24053445 10.1111/bju.12456

[8] Preminger G, Tiselius H, Assimos D, et al. 2007 Guideline for the management of ureteral calculi. J Urol 2007;178:2418–2434. 17993340 10.1016/j.juro.2007.09.107

[9] Rippel C, Nikkel L, Lin Y, et al. Residual fragments following ureteroscopic lithotripsy: Incidence and predictors on postoperative computerized tomography. J Urol 2012;188:2246–2251. 23083650 10.1016/j.juro.2012.08.040

[10] De la Rosette J, Denstedt J, Geavelete P, et al. The Clinical Research Office of the Endourological Society Ureteroscopy Global Study: Indications, complications, and outcomes in 11,885 patients. J Endourol 2014;28:131–139. 24147820 10.1089/end.2013.0436

[11] Canvasser N, Lay A, Kolitz E, Antonelli J, Pearle M. Prospective evaluation of stone free rates by computed tomography after aggressive ureteroscopy. J Urol 2017;197 (4 Suppl):MP75-12.

[12] Delarosette J, Assimos D, Desai M, et al. The Clinical Research Office of the Endourological Society Percutaneous Nephrolithotomy Global Study: Indications, complications and outcomes in 5803 patients. J Endourol 2011;25:11–17. 21247286 10.1089/end.2010.0424

[13] Li Z, Lai C, Shah A, et al. Comparative analysis of retrograde intrarenal surgery and modified ultra-mini percutaneous nephrolithotomy in management of lower pole renal stones (1.5–3.5 cm). BMC Urol 2020;20:27. 32178654 10.1186/s12894-020-00586-6 PMC7074985

[14] Pearle M, Lingeman J, Leveillee R, et al. Prospective randomized trial comparing shockwave lithotripsy and ureteroscopy for lower pole caliceal calculi 1 cm or less. J Urol 2005;173:2005–2009. 15879805 10.1097/01.ju.0000158458.51706.56

[15] Fayad A, Elsheikh M, Ghoneima W. Tubeless mini-percutaneous nephrolithotomy versus retrograde intrarenal surgery for lower calyceal stones of ≤2 cm: A prospective randomized controlled study. Arab J Urol 2016;15:36–41. 28275516 10.1016/j.aju.2016.10.002 PMC5329753

